# Machine Learning for Health: Algorithm Auditing & Quality Control

**DOI:** 10.1007/s10916-021-01783-y

**Published:** 2021-11-02

**Authors:** Luis Oala, Andrew G. Murchison, Pradeep Balachandran, Shruti Choudhary, Jana Fehr, Alixandro Werneck Leite, Peter G. Goldschmidt, Christian Johner, Elora D. M. Schörverth, Rose Nakasi, Martin Meyer, Federico Cabitza, Pat Baird, Carolin Prabhu, Eva Weicken, Xiaoxuan Liu, Markus Wenzel, Steffen Vogler, Darlington Akogo, Shada Alsalamah, Emre Kazim, Adriano Koshiyama, Sven Piechottka, Sheena Macpherson, Ian Shadforth, Regina Geierhofer, Christian Matek, Joachim Krois, Bruno Sanguinetti, Matthew Arentz, Pavol Bielik, Saul Calderon-Ramirez, Auss Abbood, Nicolas Langer, Stefan Haufe, Ferath Kherif, Sameer Pujari, Wojciech Samek, Thomas Wiegand

**Affiliations:** 1 Fraunhofer HHI, Berlin, Germany; 2 Oxford University Hospitals NHS Foundation Trust, Oxford, United Kingdom; 3 Technical Consultant (Digital Health), Thiruvananthapuram, India; 4 University of Oxford, Oxford, United Kingdom; 5 Hasso-Plattner-Institute of Digital Engineering, Potsdam, Germany; 6 Machine Learning Laboratory in Finance and Organizations, Universidade de Brasília, Brasília, Brazil; 7 World Development Group Inc, Bethesda, MD USA; 8 Johner Institute, Konstanz, Germany; 9 Makerere University, Kampala, Uganda; 10 Siemens Healthineers, Erlangen, Germany; 11 University of Milano-Bicocca, Milan, Italy; 12 Philips, New Kensington, USA; 13 Office of the Auditor General of Norway, Oslo, Norway; 14 University Hospitals Birmingham NHS Foundation Trust & Academic Unit of Ophthalmology, Institute of Inflammation and Ageing, College of Medical and Dental Sciences, University of Birmingham, Birmingham, United Kingdom; 15 Bayer AG, Berlin, Germany; 16 minoHealth AI Labs, Accra, Ghana; 17 Information Systems Department, College of Computer and Information Sciences, King Saud University, Riyadh, Saudi Arabia; 18 Digital Health and Innovation Department, Science Division, World Health Organization, Winterthur, Switzerland; 19 University College London, London, United Kingdom; 20 Open Regulatory, Bonn, Germany; 21 MIOTIFY LTD, London, United Kingdom; 22 IEC TC62 and Siemens Healthineers, Erlangen, Germany; 23 Helmholtz Zentrum München, Neuherberg, Germany; 24 Oral Diagnostics Digital Health Health Services Research, Charité-Universitätsmedizin, Berlin, Germany; 25 Dotphoton AG, Zug, Switzerland; 26 Department of Global Health, University of Washington, Washington, USA; 27 LatticeFlow & ETH Zurich, Zürich, Switzerland; 28 De Montfort University & Instituto Tecnologico de Costa Rica, Cartago, Costa Rica; 29 Robert Koch Institut, Berlin, Germany; 30 Department of Psychology, University of Zurich, Zürich, Switzerland; 31 Technische Universität Berlin, Berlin, Germany; 32 Laboratory for Research in Neuroimaging, Department of Clinical Neuroscience, Lausanne University Hospital and University of Lausanne, Lausanne, Switzerland

**Keywords:** Machine learning, Artificial intelligence, Algorithm, Health, Auditing, Quality control

## Abstract

Developers proposing new machine learning for health (ML4H) tools often pledge to match or even surpass the performance of existing tools, yet the reality is usually more complicated. Reliable deployment of ML4H to the real world is challenging as examples from diabetic retinopathy or Covid-19 screening show. We envision an integrated framework of algorithm auditing and quality control that provides a path towards the effective and reliable application of ML systems in healthcare. In this editorial, we give a summary of ongoing work towards that vision and announce a call for participation to the special issue  *Machine Learning for Health: Algorithm Auditing & Quality Control* in this journal to advance the practice of ML4H auditing.

**Supplementary Information:**

The online version contains supplementary material available at 10.1007/s10916-021-01783-y.

## Introduction

Machine learning (ML) technology promises to automate, speed up or improve medical processes. A large number of institutions and companies are ambitiously working on fulfilling this promise spanning tasks such as medical image classification [1], segmentation [2] or reconstruction [3], protein structure prediction [4] and electrocardiography interpretation [5], among others^1^. However, the deployment of machine learning for health (ML4H) tools into real-world applications has been slow because existing approval processes [6] may not account for the particular failure modes and risks that accompany (ML) technology [7–11]. Certain changes to image data that may not change the decision of a human expert can completely alter the output of an image classification [12] or regression [13, 14] model. Model performance estimates are often not valid for the types of varying input distribution that can occur during real world deployment [15–17]. The decision heuristics a model learns can differ from the heuristics we may expect a human to use [1, 18–20], and model predictions may come with ill-calibrated statements of confidence [21–23] or no estimate of uncertainty altogether [24]. Developers proposing new ML4H technologies sometimes promise to match or even surpass the performance of existing methods [25] yet the reality is often more complicated. Classical ML performance evaluation does not automatically translate to clinical utility as examples from large diabetic retinopathy projects [26] or Covid-19 diagnosis illustrate [27]. The reliable and integrated management of these risks remains an open scientific and practical hurdle.

In order to overcome this hurdle, we envision a framework of algorithm auditing and quality control that provides a path towards the effective and reliable application of ML systems in healthcare. In this editorial we give a brief summary of ongoing work towards that vision from our open collective of collaborators. Many of the considerations presented here originate from a consensus finding effort by the International Telecommunication Union (ITU) and World Health Organization (WHO) which started in 2018 as the Focus Group on Artificial Intelligence for Health (FG-AI4H) [28].

We are convinced that success on this path heavily depends on practical feedback. Auditing processes that are developed on paper have to be put to the test to ensure that they translate to utility in the actual auditing practice [29]. That is why we are introducing the special issue *Machine Learning for Health: Algorithm Auditing & Quality Control* in this journal (see the Call for Participation for more details^2^). The special issue will provide a platform for the submission, discussion and publication of audit methods and reports. The resulting compendium is intended to be a useful resource for users, developers, vendors and auditors of ML4H systems to manage and mitigate their particular risks.

## ML4H Algorithm Auditing & Quality Control

**Fig. 1:**
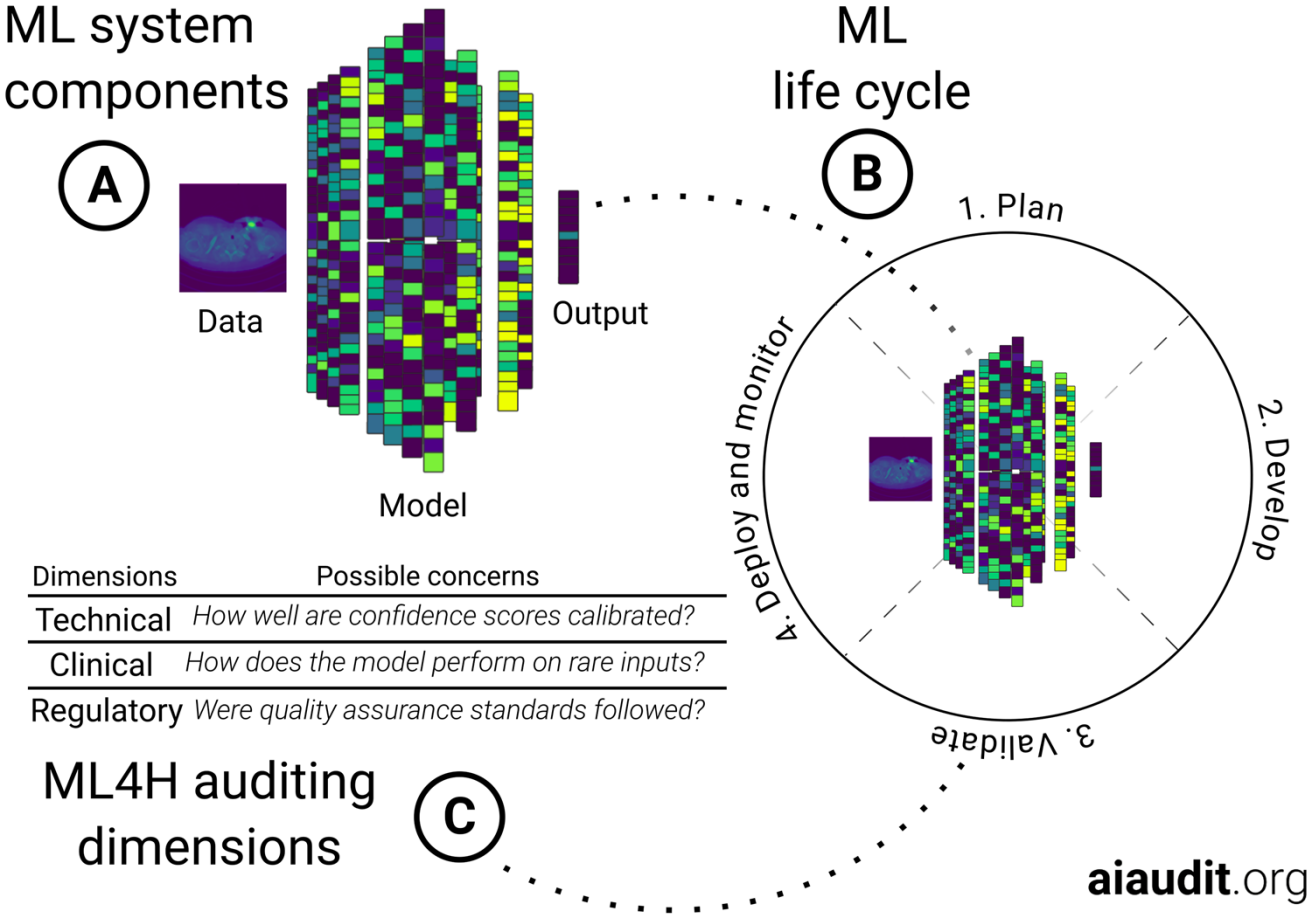
Process overview. **A**: Most ML tools share a set of core components comprising data, a ML-model and its outputs **B**: The typical ML life cycle goes through stages of planning, development, validation and, potentially, deployment under appropriate monitoring **C**: An ML4H audit is carried out with respect to a dynamic set of technical, clinical and regulatory considerations that depend on the concrete ML technology and the intended use of the tool

From a bird’s eye view, many ML tools share a set of core components comprising data, an ML-model and its outputs, as visualized in Fig. 1A. The typical ML product life cycle goes through stages of planning, development, validation and, potentially, deployment under appropriate monitoring (see Fig. 1B). Feedback loops between stages, for example from product validation back to development, are commonplace^3^.

An audit entails a detailed assessment of an ML4H tool at one or more of the ML life cycle steps. It can be carried out to anticipate, monitor, or retrospectively review operations of the tool [30, 31]. The audit output should consist of a comprehensive standardized report that can be used by different stakeholders to efficiently communicate the tool’s strengths and limitations [29]. We envision a process by which an independent body, for example appointed by a government, carries out the audit using the methods and tools outlined below. Further, they can also be used by manufacturers and researchers themselves to carry out internal quality control [32]. In either scenario, the assessment is carried out with respect to a dynamic set of technical, clinical and regulatory considerations (see Fig. 1C) that depend on the concrete ML technology and the intended use of the tool. Audit teams should thus comprise expertise in all these dimensions and have to be able to synthesize related requirements across disciplines. In the following, we list a selection of considerations for all three of these auditing dimensions, tools that can be used to aid the auditing process as well as the role so called trial audits can play in advancing ML4H quality control.

### Auditing Dimensions

The **technical validation** of an ML4H tool comprises the application of data and ML model quality assessment methods to detect possible failure modes in the model’s behavior. These include model-oriented metrics, such as predictive performance, robustness [33, 34], interpretability [1, 35], disparity [36] or uncertainty [13, 24, 37] but also data-oriented metrics related to sample size determination [38], sparseness [39], bias [40] distribution mismatch [41, 42] and label quality [7]. Rigorous statistical analysis of the model metrics is a common pitfall in both research and industry, and thus plays an important role during technical validation [43]. FG-AI4H has formulated a standardized quality assessment framework based on existing good practices [44–46] and provides practical guidance and examples for performing technical validation audits on three ML4H tools [29].

**Clinical Evaluation** comprises an “ongoing procedure to collect, appraise and analyse clinical data pertaining to a medical device and to analyse whether there is sufficient clinical evidence to confirm compliance with relevant essential requirements for safety and performance when using the device according to the manufacturer’s instructions for use” [47]. The EQUATOR-network, including STARD-AI [48], CONSORT-AI [49] and SPIRIT-AI [50], as well as different scientific journals and associations [51–54], have developed guidelines for the design, implementation, reporting and evaluation of AI interventions in various study designs. Key concerns are whether the ML4H tool delivers utility in clinical pathways, how cost-effective the clinician-tool interaction is [55] and whether it provides the desired benefits for the intended users [56]. To demonstrate reliable performance, it is important to look beyond common machine learning performance statistics such as accuracy and to evaluate in addition whether the ML4H tool is suited to the clinical setting in which it will be used; for example, whether the training and test data represent patient populations that are similar to the intended use population [7, 57] and whether the output translates to medically meaningful parameters [58].

**Regulatory Assessment** comprises the systematic evaluation of ML4H tools with respect to the applicable regulatory requirements found in laws (MDR [59], IVDR [60], 21 CFR [61], among others), to international standards (such as IEC 62304 [62], IEC 62366-1 [63] and ISO 14971 [64]), to guidelines by regulatory bodies (for example FDA [65], IMDRF [66]) or to guidelines and drafts by other organizations (for example AAMI [67] or European Commission [68]). Such guidance is of practical concern for stakeholders in the ML4H ecosystem including manufacturers (e.g. product managers, developers, developers and data scientists, quality and regulatory affairs managers) and for regulatory bodies (authorities, notified bodies). The FG-AI4H has identified and critically reviewed general yet fundamental regulatory considerations related to ML4H. This overview of regulatory considerations assessment have been converted into specific and verifiable requirements and subsequently published as a comprehensive assessment checklist entitled “Good practices for health applications of machine learning: Considerations for manufacturers and regulators” [45] which covers the entire life cycle outlined in 1B at a higher resolution. It includes checklist items which should be given high priority in the presence of limited time - an important practical constraint for real-world audits. Examples and comments give further guidance to users. New regulatory developments, such as predetermined change control plans [69], imply faster software update cycles and potentially more frequent audits. Hence, good tooling can become an important means to make effective as well as efficient audits possible.

### Auditing Tools

The auditing process can be supported by appropriate tools to make it more targeted and time-efficient. This can include process and requirements descriptions, as mentioned above [44, 45, 56], which help to manage dynamic workflows that may vary by use case and ML technology. It also includes reporting templates to present the audit results in a standardized way for the communication between different stakeholders. [29, 70]. In addition, the nature of ML4H tools, as primarily software that interacts with data, lends itself to the application of test automation and simulations for the purpose of auditing. This requires software tools which can handle custom evaluation scripts, the flexible processing of different ML4H model formats and data modalities as well as security protocols that protect intellectual property and sensitive patient information [71]. We are working with open source frameworks such as EvalAI [72] and MLflow [73] to develop solutions for automated auditing^4^, federated auditing in remote teams^5^ and automated report creation. Our first demo platform is available via http://health.aiaudit.org/6 and hosted on ITU provisioned infrastructure. While quantitative performance measures can already be provided, it is essential to also offer qualitative measures. This is realized by requiring the users to fill out a standardized questionnaire [74]. Quantitative and qualitative performance results are then provided to the users as a comprehensive and standardized report card [70].

### Trial Audits

We are convinced that success on the path towards a framework for algorithm auditing and quality control depends heavily on practical feedback. The development and refinement of auditing processes should routinely be accompanied by trial audits. In trial audits, draft processes and standards are applied to ML4H tools. The purpose of such an exercise is to ensure that auditing processes developed on paper translate to utility in actual auditing practice [29]. In order to facilitate the implementation of trial audits, we are introducing the special issue *Machine Learning for Health: Algorithm Auditing & Quality Control* in this journal. We welcome contributions pertaining to methods, tools, reports or open challenges in ML4H auditing.

## Outlook

The materials summarized above bear testimony to the initial progress that has been made towards the creation of frameworks for ML4H algorithm auditing and quality control. Nevertheless, new challenges emerge as we collectively pull at the complex fabric that ML4H systems are.

From the perspective of technical validation, the identification of factors which bias or deteriorate algorithmic performance is often constrained by the absence of relevant metadata. For example, the measurement device types (and related acquisition parameters) used to produce the validation inputs should be available in order to validate if the model performance is robust under device type changes. This problem can be alleviated by identifying and routinely recording this information during data acquisition.

For clinical evaluation, future considerations include extending and refining the specific requirements related to how the clinical effectiveness of a tool should be monitored after implementation of the algorithm and with ongoing monitoring [59]. This also requires agreement over the clear and clinically useful procedures to obtain ground truth annotations. It might be necessary to refine the ML algorithm to the target population, if demographics or clinical character are different from training settings or if medical guidelines for diagnostics or treatment have changed [75]. Therefore, in order for these insights to be effective it is imperative that auditors exhibit a solid understanding of the training data, ML algorithm, independent test data and evaluation metrics specific to the intended use.

A challenge for regulatory assessment is that standardization organizations, notified bodies and manufacturers need to efficiently formulate and parse applicable regulatory requirements for each individual ML4H tool. Comprehensive assessment checklists [45, 51] can help with that task. However, more support is needed in terms of workflow management and assisting tools if we consider the limited time and budgets which professional auditors have at their disposal. Future regulatory checklists should allow for interactive selection of use-case specific sub-checklists, an automated audit report creation, a issue of standard minimum test cases as well as accompanying glossaries and education materials for auditors. We also have to ensure that protocols are in place which translate the audit insights to actual improvements in the ML4H tool. Managing the risks presented by the exciting advances of AI in healthcare is a formidable undertaking, but with collaborative pooling of expertise and resources we believe we can rise to the task.

## Supplementary Information

Below is the link to the electronic supplementary material.

Supplementary file1 (PDF 241 KB)

## Footnotes

1 The larger machine learning community maintains a good overview of tasks, benchmarks and state-of-the-art methods at https://paperswithcode.com/.

2 In the supplement and at this address https://aiaudit.org/joms/

3 Both representations A and B in 1 are high level abstractions. A granular taxonomy of ML tools or their life cycles is beyond the scope of this editorial. We refer the interested reader to [76] and our documentation [45] for an in-depth treatment.

4 https://github.com/aiaudit-org/health-aiaudit-public

5 https://github.com/aiaudit-org/amazon-sagemaker-mlflow-fargate

6 You are welcome to reach out to any of the contributors https://aiaudit.org/contributors/ for information on how to join the efforts.

## References

[1] Hägele M, Seegerer P, Lapuschkin S, Bockmayr M, Samek W, Klauschen F, Müller K-R, Binder A (2020). Resolving challenges in deep learning-based analyses of histopathological images using explanation methods. Scientific Reports.

[2] Zhou, Z., Siddiquee, M. M. R., Tajbakhsh, N., and Liang, J. Unet++: A nested u-net architecture for medical image segmentation. In *Deep learning in medical image analysis and multimodal learning for clinical decision support*. Springer, 2018, pp. 3–11. 10.1007/978-3-030-00889-5_1 PMC7329239 32613207

[3] Bubba TA, Kutyniok G, Lassas M, März M, Samek W, Siltanen S, Srinivasan V (2019). Learning the invisible: a hybrid deep learning-shearlet framework for limited angle computed tomography. Inverse Problems.

[4] Senior AW, Evans R, Jumper J, Kirkpatrick J, Sifre L, Green T, Qin C, Žídek A, Nelson AW, Bridgland A (2020). Improved protein structure prediction using potentials from deep learning. Nature.

[5] Wagner P, Strodthoff N, Bousseljot R-D, Kreiseler D, Lunze FI, Samek W, Schaeffter T (2020). Ptb-xl, a large publicly available electrocardiography dataset. Scientific Data.

[6] Wu E, Wu K, Daneshjou R, Ouyang D, Ho DE, Zou J (2021). How medical ai devices are evaluated: limitations and recommendations from an analysis of fda approvals. Nature Medicine.

[7] Cabitza, F., Campagner, A., and Sconfienza, L. M. As if sand were stone. new concepts and metrics to probe the ground on which to build trustable ai. *BMC Medical Informatics and Decision Making 20*, 1 (2020), 1–21. 10.1186/s12911-020-01224-9 PMC7488864 32917183

[8] D’Amour, A., Heller, K., Moldovan, D., Adlam, B., Alipanahi, B., Beutel, A., Chen, C., Deaton, J., Eisenstein, J., Hoffman, M. D., et al. Underspecification presents challenges for credibility in modern machine learning. arXiv preprint arXiv:2011.03395 (2020).

[9] Gilmer, J., Ford, N., Carlini, N., and Cubuk, E. Adversarial examples are a natural consequence of test error in noise. In *International Conference on Machine Learning* (2019), PMLR, pp. 2280–2289.

[10] Raji, I. D., Smart, A., White, R. N., Mitchell, M., Gebru, T., Hutchinson, B., Smith-Loud, J., Theron, D., and Barnes, P. Closing the ai accountability gap: defining an end-to-end framework for internal algorithmic auditing. In *Proceedings of the 2020 Conference on Fairness, Accountability, and Transparency* (2020), pp. 33–44.

[11] Recht, B., Roelofs, R., Schmidt, L., and Shankar, V. Do imagenet classifiers generalize to imagenet? In *International Conference on Machine Learning* (2019), PMLR, pp. 5389–5400. http://www.bmj.com/lookup/doi/10.1136/bmj.m3210

[12] Szegedy, C., Zaremba, W., Sutskever, I., Bruna, J., Erhan, D., Goodfellow, I., and Fergus, R. Intriguing properties of neural networks. arXiv preprint arXiv:1312.6199 (2013).

[13] Macdonald, J., März, M., Oala, L., and Samek, W. Interval neural networks as instability detectors for image reconstructions. In *Bildverarbeitung für die Medizin 2021* (Wiesbaden, 2021), C. Palm, T. M. Deserno, H. Handels, A. Maier, K. Maier-Hein, and T. Tolxdorff, Eds., Springer Fachmedien Wiesbaden, pp. 324–329.

[14] Oala, L., Heiß, C., Macdonald, J., März, M., Kutyniok, G., and Samek, W. Detecting failure modes in image reconstructions with interval neural network uncertainty. *International Journal of Computer Assisted Radiology and Surgery* (2021), 1–9. https://arxiv.org/abs/2003.11566 10.1007/s11548-021-02482-2 PMC8616888 34480723

[15] Hendrycks, D., Basart, S., Mu, N., Kadavath, S., Wang, F., Dorundo, E., Desai, R., Zhu, T., Parajuli, S., Guo, M., et al. The many faces of robustness: A critical analysis of out-of-distribution generalization. arXiv preprint arXiv:2006.16241 (2020).

[16] Taori, R., Dave, A., Shankar, V., Carlini, N., Recht, B., and Schmidt, L. Measuring robustness to natural distribution shifts in image classification. arXiv preprint arXiv:2007.00644 (2020).

[17] Willis, K., and Oala, L. Post-hoc domain adaptation via guided data homogenization. *CoRR abs/2104.03624* (2021). https://arxiv.org/abs/2104.03624

[18] Lapuschkin S, Wäldchen S, Binder A, Montavon G, Samek W, Müller K-R (2019). Unmasking clever hans predictors and assessing what machines really learn. Nature Communications.

[19] Nalisnick, E., Matsukawa, A., Teh, Y. W., Gorur, D., and Lakshminarayanan, B. Do deep generative models know what they don’t know? arXiv preprint arXiv:1810.09136 (2018).

[20] Neves I, Folgado D, Santos S, Barandas M, Campagner A, Ronzio L, Cabitza F, Gamboa H (2021). Interpretable heartbeat classification using local model-agnostic explanations on ecgs. Computers in Biology and Medicine.

[21] Calderon-Ramirez S, Yang S, Moemeni A, Colreavy-Donnelly S, Elizondo DA, Oala L, Rodríguez-Capitán J, Jiménez-Navarro M, López-Rubio E, Molina-Cabello MA (2021). Improving uncertainty estimation with semi-supervised deep learning for covid-19 detection using chest x-ray images. IEEE Access.

[22] Guo, C., Pleiss, G., Sun, Y., and Weinberger, K. Q. On calibration of modern neural networks. In *International Conference on Machine Learning* (2017), PMLR, pp. 1321–1330.

[23] Minderer, M., Djolonga, J., Romijnders, R., Hubis, F., Zhai, X., Houlsby, N., Tran, D., and Lucic, M. Revisiting the calibration of modern neural networks, 2021.

[24] Kendall, A., and Gal, Y. What uncertainties do we need in bayesian deep learning for computer vision? In *Advances in Neural Information Processing Systems* (2017), I. Guyon, U. V. Luxburg, S. Bengio, H. Wallach, R. Fergus, S. Vishwanathan, and R. Garnett, Eds., vol. 30, Curran Associates, Inc. https://proceedings.neurips.cc/paper/2017/file/2650d6089a6d640c5e85b2b88265dc2b-Paper.pdf

[25] Roberts M, Driggs D, Thorpe M, Gilbey J, Yeung M, Ursprung S, Aviles-Rivero AI, Etmann C, McCague C, Beer L (2021). Common pitfalls and recommendations for using machine learning to detect and prognosticate for covid-19 using chest radiographs and ct scans. Nature Machine Intelligence.

[26] Heaven, W. D. Google’s medical ai was super accurate in a lab. real life was a different story. | mit technology review. https://www.technologyreview.com/2020/04/27/1000658/google-medical-ai-accurate-lab-real-life-clinic-covid-diabetes-retina-disease/. (Accessed on 06/10/2021).

[27] Oakden-Rayner, L. Ct scanning is just awful for diagnosing covid-19 – luke oakden-rayner. https://lukeoakdenrayner.wordpress.com/2020/03/23/ct-scanning-is-just-awful-for-diagnosing-covid-19/. (Accessed on 06/10/2021).

[28] Wiegand T, Krishnamurthy R, Kuglitsch M, Lee N, Pujari S, Salathé M, Wenzel M, Xu S (2019). Who and itu establish benchmarking process for artificial intelligence in health. The Lancet.

[29] Oala, L., Fehr, J., Gilli, L., Balachandran, P., Leite, A. W., Calderon-Ramirez, S., Li, D. X., Nobis, G., Alvarado, E. A. M. n., Jaramillo-Gutierrez, G., Matek, C., Shroff, A., Kherif, F., Sanguinetti, B., and Wiegand, T. Ml4h auditing: From paper to practice. In *Proceedings of the Machine Learning for Health NeurIPS Workshop* (2020), vol. 136, PMLR, pp. 280–317.

[30] Koshiyama, A., Kazim, E., Treleaven, P., Rai, P., Szpruch, L., Pavey, G., Ahamat, G., Leutner, F., Goebel, R., Knight, A., et al. Towards algorithm auditing: A survey on managing legal, ethical and technological risks of ai, ml and associated algorithms. 10.1098/rsos.230859 PMC11285902 39076787

[31] Shneiderman B (2016). Opinion: The dangers of faulty, biased, or malicious algorithms requires independent oversight. Proceedings of the National Academy of Sciences.

[32] Ryan, J. R. Software product quality assurance. In *Proceedings of the June 7-10, 1982, National Computer Conference* (New York, NY, USA, 1982), AFIPS ’82, Association for Computing Machinery, p. 393–398. 10.1145/1500774.1500823

[33] Carlini, N., and Wagner, D. Towards evaluating the robustness of neural networks. In *2017 ieee symposium on security and privacy (sp)* (2017), IEEE, pp. 39–57.

[34] Hendrycks, D., and Dietterich, T. Benchmarking neural network robustness to common corruptions and perturbations. arXiv preprint arXiv:1903.12261 (2019).

[35] Samek W, Montavon G, Lapuschkin S, Anders CJ, Müller K-R (2021). Explaining deep neural networks and beyond: A review of methods and applications. Proceedings of the IEEE.

[36] Saleiro, P., Kuester, B., Hinkson, L., London, J., Stevens, A., Anisfeld, A., Rodolfa, K. T., and Ghani, R. Aequitas: A bias and fairness audit toolkit. arXiv preprint arXiv:1811.05577 (2018).

[37] Oala, L., Heiß, C., MacDonald, J., März, M., Samek, W., and Kutyniok, G. Interval neural networks: Uncertainty scores. *CoRR abs/2003.11566* (2020).

[38] Balki I, Amirabadi A, Levman J, Martel AL, Emersic Z, Meden B, Garcia-Pedrero A, Ramirez SC, Kong D, Moody AR (2019). Sample-size determination methodologies for machine learning in medical imaging research: a systematic review. Canadian Association of Radiologists Journal.

[39] Mendez, M., Calderon-Ramirez, S., and Tyrrell, P. N. Using cluster analysis to assess the impact of dataset heterogeneity on deep convolutional network accuracy: A first glance. In *Latin American High Performance Computing Conference* (2019), Springer, pp. 307–319.

[40] Noseworthy, P. A., Attia, Z. I., Brewer, L. C., Hayes, S. N., Yao, X., Kapa, S., Friedman, P. A., and Lopez-Jimenez, F. Assessing and mitigating bias in medical artificial intelligence: the effects of race and ethnicity on a deep learning model for ecg analysis. *Circulation: Arrhythmia and Electrophysiology 13*, 3 (2020), e007988. 10.1161/CIRCEP.119.007988 PMC7158877 32064914

[41] Mårtensson G, Ferreira D, Granberg T, Cavallin L, Oppedal K, Padovani A, Rektorova I, Bonanni L, Pardini M, Kramberger MG (2020). The reliability of a deep learning model in clinical out-of-distribution mri data: a multicohort study. Medical Image Analysis.

[42] Ramírez, S. C., and Oala, L. More than meets the eye: Semi-supervised learning under non-iid data. *CoRR abs/2104.10223* (2021). https://arxiv.org/abs/2104.10223

[43] Parmar C, Barry JD, Hosny A, Quackenbush J, Aerts HJ (2018). Data analysis strategies in medical imaging. Clinical cancer research.

[44] FG-AI4H. Data and artificial intelligence assessment methods (daisam) reference. *Reference document DEL 7.3 on FG-AI4H server* (2020). https://extranet.itu.int/sites/itu-t/focusgroups/ai4h/SitePages/Home.aspx

[45] Johner, C., Balachandran, P., Oala, L., Lee, A. Y., Werneck Leite, A., Murchison, A., Lin, A., Molnar, C., Rumball-Smith, J., Baird, P., Goldschmidt, P. G., Quartarolo, P., Xu, S., Piechottka, S., and Hornberger, Z. Good practices for health applications of machine learning: Considerations for manufacturers and regulators. In *ITU/WHO Focus Group on Artificial Intelligence for Health (FG-AI4H) - Meeting K* (2021), L. Oala, Ed., vol. K, ITU. https://extranet.itu.int/sites/itu-t/focusgroups/ai4h/SitePages/Home.aspx

[46] The Supreme Audit Institutions of Finland, Germany, the Netherlands, Norway and the UK. Auditing machine learning algorithms. https://auditingalgorithms.net/, 2020. (Accessed on 07/02/2021).

[47] EUROPEAN-COMMISSION. Meddev 2.7/1 revision 4, clinical evaluation: a guide for manufacturers and notified bodies. https://ec.europa.eu/docsroom/documents/17522/attachments/1/translations/en/renditions/native, 2016. (Accessed on 07/01/2021).

[48] Sounderajah V, Ashrafian H, Aggarwal R, De Fauw J, Denniston AK, Greaves F, Karthikesalingam A, King D, Liu X, Markar SR, McInnes MD, Panch T, Pearson-Stuttard J, Ting DS, Golub RM, Moher D, Bossuyt PM, Darzi A (2020). Developing specific reporting guidelines for diagnostic accuracy studies assessing AI interventions: The STARD-AI Steering Group. Nature Medicine.

[49] Liu, X., Cruz Rivera, S., Moher, D., Calvert, M., Denniston, A. K., Spirit-ai, T., and Group, C.-a. W. CONSORT-AI extension. *Nature Medicine 26*, September (2020), 1364–1374.

[50] Rivera SC, Liu X, Chan A-W, Denniston AK, Calvert MJ (2020). Guidelines for clinical trial protocols for interventions involving artificial intelligence: the SPIRIT-AI Extension. Bmj.

[51] Cabitza, F., and Campagner, A. The need to separate the wheat from the chaff in medical informatics. *International Journal of Medical Informatics* (2021), 104510. 10.1016/j.ijmedinf.2021.104510 34108105

[52] Hernandez-Boussard T, Bozkurt S, Ioannidis JP, Shah NH (2020). Minimar (minimum information for medical ai reporting): developing reporting standards for artificial intelligence in health care. Journal of the American Medical Informatics Association.

[53] Schwendicke F, Singh T, Lee J-H, Gaudin R, Chaurasia A, Wiegand T, Uribe S, Krois J (2021). Artificial intelligence in dental research: Checklist for authors, reviewers, readers. Journal of Dentistry.

[54] Scott I, Carter S, Coiera E (2021). Clinician checklist for assessing suitability of machine learning applications in healthcare. BMJ Health & Care Informatics.

[55] Schwendicke F, Rossi J, Göstemeyer G, Elhennawy K, Cantu A, Gaudin R, Chaurasia A, Gehrung S, Krois J (2021). Cost-effectiveness of artificial intelligence for proximal caries detection. Journal of Dental Research.

[56] FG-AI4H. Clinical evaluation of ai for health. *Reference document DEL 7.4 on FG-AI4H server* (2021). https://extranet.itu.int/sites/itu-t/focusgroups/ai4h/SitePages/Home.aspx

[57] Kaushal, A., Altman, R., and Langlotz, C. Geographic Distribution of US Cohorts Used to Train Deep Learning Algorithms. *JAMA 324*, 12 (09 2020), 1212–1213. 10.1001/jama.2020.12067 10.1001/jama.2020.12067 PMC7509620 32960230

[58] Nagendran M, Chen Y, Lovejoy CA, Gordon AC, Komorowski M, Harvey H, Topol EJ, Ionnidis JPA, Collins GS, Maruthappu M (2020). Artificial intelligence versus clinicians: systematic review of design, reporting standards, and clains of deep learning studies. British Medical Journal.

[59] EU. Regulation (eu) 2017/746 of the european parliament and of the council on medical devices, (2017). https://eur-lex.europa.eu/eli/reg/2017/745/oj

[60] EU. Regulation (eu) 2017/746 of the european parliament and of the council on in vitro diagnostic medical devices, (2017). https://eur-lex.europa.eu/eli/reg/2017/746/oj

[61] FDA. Code of federal regulations, title 21 on foods and drugs. https://www.ecfr.gov/cgi-bin/text-idx?SID=cc74806513924f0197b7809c8efbefc8&mc=true&tpl=/ecfrbrowse/Title21/21tab_02.tpl

[62] IEC. Medical device software – software life cycle processes – amendment 1 (2015). https://www.iso.org/standard/64686.html

[63] IEC. Medical devices – part 1: Application of usability engineering to medical devices – amendment 1 (2020). https://www.iso.org/standard/73007.html

[64] ISO. Medical devices – application of risk management to medical devices (2019). https://www.iso.org/standard/72704.html

[65] FDA. Fda guidance documents. https://www.fda.gov/regulatory-information/search-fda-guidance-documents

[66] IMDRF. Documents by international medical device regulators forum. http://www.imdrf.org/documents/documents.asp

[67] AAMI. Techical report (tr) 57 principals for medical device security - risk management. https://store.aami.org/s/store#/store/browse/detail/a152E000006j60WQAQ

[68] EUROPEAN-COMMISSION. Eur-lex - 52021pc0206 - en - eur-lex. https://eur-lex.europa.eu/legal-content/EN/TXT/?uri=CELEX:52021PC0206, 2021. (Accessed on 07/01/2021).

[69] US-FDA. Aiml\_samd\_action\_plan. https://www.fda.gov/media/145022/download?utm_medium=email&utm_source=govdelivery, 2021. (Accessed on 07/01/2021).

[70] Verks, B., and Oala, L. Daisam audit reporting template. In *ITU/WHO Focus Group on Artificial Intelligence for Health (FG-AI4H) - Meeting J* (2020), vol. J, ITU. https://extranet.itu.int/sites/itu-t/focusgroups/ai4h/SitePages/Home.aspx

[71] FG-AI4H. Data sharing practices. *Reference document DEL 5.6 on FG-AI4H server* (2021). https://extranet.itu.int/sites/itu-t/focusgroups/ai4h/SitePages/Home.aspx

[72] Yadav, D., Jain, R., Agrawal, H., Chattopadhyay, P., Singh, T., Jain, A., Singh, S., Lee, S., and Batra, D. Evalai: Towards better evaluation systems for AI agents. *CoRR abs/1902.03570* (2019). http://arxiv.org/abs/1902.03570

[73] Chen, A., Chow, A., Davidson, A., DCunha, A., Ghodsi, A., Hong, S. A., Konwinski, A., Mewald, C., Murching, S., Nykodym, T., Ogilvie, P., Parkhe, M., Singh, A., Xie, F., Zaharia, M., Zang, R., Zheng, J., and Zumar, C. Developments in mlflow: A system to accelerate the machine learning lifecycle. In *Proceedings of the Fourth International Workshop on Data Management for End-to-End Machine Learning* (New York, NY, USA, 2020), DEEM’20, Association for Computing Machinery. 10.1145/3399579.3399867

[74] FG-AI4H. Model questionnaire. *Reference document J-038 on FG-AI4H server* (2020). https://extranet.itu.int/sites/itu-t/focusgroups/ai4h/SitePages/Home.aspx

[75] Kelly CJ, Karthikesalingam A, Suleyman M, Corrado G, King D (2019). Key challenges for delivering clinical impact with artificial intelligence. BMC Medicine.

[76] Hardt, M., and Recht, B. *Patterns, predictions, and actions: A story about machine learning*. https://mlstory.org (2021).

