# Supplementary material for: Machine Learning for Health: Algorithm Auditing & Quality Control
Source: J Med Syst. 2021 Nov 2;45(12):105. doi: 10.1007/s10916-021-01783-y (PMC8562935; doi:10.1007/s10916-021-01783-y)
Supplement: Supplementary file 1 — Supplementary file1 (PDF 241 KB) [file 10916_2021_1783_MOESM1_ESM.pdf]

Call for Papers

Journal of Medical Systems
Special Collection

Machine Learning for Health: Algorithm Auditing & Quality Control

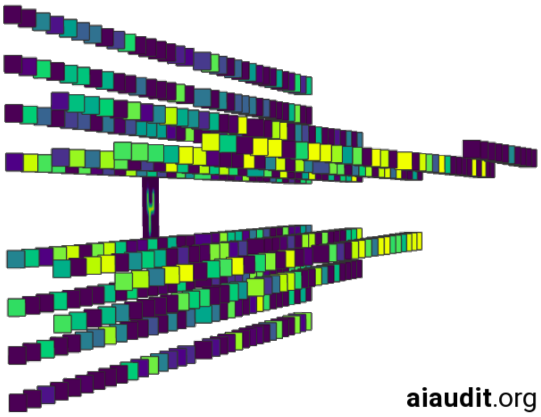

We invite researchers and practitioners in the fields of machine learning, medicine, regulation and quality management to submit work to the special collection *Machine Learning for Health: Algorithm Auditing & Quality Control*. The scope comprises machine learning for health (ML4H) trial audit reports as well as work related to methods, tools or open challenges in ML4H auditing. The resulting compendium is intended as a useful resource of examples and guidance for users, developers, vendors and auditors of ML4H systems to better manage and mitigate their particular risks. Manuscripts which treat topics from an integrated perspective across disciplines are encouraged. Below you can find a list of good example papers from each category.

Audit reports

– External Validation of a Widely Implemented Proprietary Sepsis Prediction Model in Hospitalized Patients [6]
– ML4H Auditing: From Paper to Practice [5]

Methods

– Benchmarking Neural Network Robustness to Common Corruptions and Perturbations [4]
– Panning for gold:'model-X'knockoffs for high dimensional controlled variable selection [1]

Tools

– EvalAI: Towards Better Evaluation Systems for AI Agents [7]
– Developments in MLflow: A System to Accelerate the Machine Learning Lifecycle [2]

Open challenges

- Understanding deep learning (still) requires rethinking generalization [8]
- Underspecification presents challenges for credibility in modern machine learning [3]

Manuscripts should be submitted as TODO: specify the paper category through the JOMS Editorial Manager[1]. Please use the document template provided by JOMS[2][3].

In case of questions you can reach the Editorial Team at TODO: contact for questions.

We are looking forward to receiving your interesting contributions.

On behalf of the Editorial Team
Jesse Ehrenfeld, Liz Hoffman, Luis Oala
Cyberspace, August 16, 2021

References

1. Candès, E., Fan, Y., Janson, L., Lv, J.: Panning for gold:'model-x'knockoffs for high dimensional controlled variable selection. Journal of the Royal Statistical Society: Series B (Statistical Methodology) **80**(3), 551–577 (2018)
2. Chen, A., Chow, A., Davidson, A., DCunha, A., Ghodsi, A., Hong, S.A., Konwinski, A., Mewald, C., Murching, S., Nykodym, T., Ogilvie, P., Parkhe, M., Singh, A., Xie, F., Zaharia, M., Zang, R., Zheng, J., Zumar, C.: Developments in mlflow: A system to accelerate the machine learning lifecycle. In: Proceedings of the Fourth International Workshop on Data Management for End-to-End Machine Learning, DEEM'20. Association for Computing Machinery, New York, NY, USA (2020). DOI 10.1145/3399579.3399867. URL `https://doi.org/10.1145/3399579.3399867`
3. D'Amour, A., Heller, K., Moldovan, D., Adlam, B., Alipanahi, B., Beutel, A., Chen, C., Deaton, J., Eisenstein, J., Hoffman, M.D., et al.: Underspecification presents challenges for credibility in modern machine learning. arXiv preprint arXiv:2011.03395 (2020)
4. Hendrycks, D., Dietterich, T.: Benchmarking neural network robustness to common corruptions and perturbations. In: International Conference on Learning Representations (2018)
5. Oala, L., Fehr, J., Gilli, L., Balachandran, P., Leite, A.W., Calderon-Ramirez, S., Li, D.X., Nobis, G., Alvarado, E.A.M.n., Jaramillo-Gutierrez, G., Matek, C., Shroff, A., Kherif, F., Sanguinetti, B., Wiegand, T.: Ml4h auditing: From paper to practice. In: Proceedings of the Machine Learning for Health NeurIPS Workshop, vol. 136, pp. 280–317. PMLR (2020)
6. Wong, A., Otles, E., Donnelly, J.P., Krumm, A., McCullough, J., DeTroyer-Cooley, O., Pestrue, J., Phillips, M., Konye, J., Penoza, C., Ghous, M., Singh, K.: External Validation of a Widely Implemented Proprietary Sepsis Prediction Model in Hospitalized Patients. JAMA Internal Medicine **181**(8), 1065–1070 (2021). DOI 10.1001/jamainternmed.2021.2626. URL `https://doi.org/10.1001/jamainternmed.2021.2626`
7. Yadav, D., Jain, R., Agrawal, H., Chattopadhyay, P., Singh, T., Jain, A., Singh, S., Lee, S., Batra, D.: Evalai: Towards better evaluation systems for AI agents. CoRR **abs/1902.03570** (2019). URL `http://arxiv.org/abs/1902.03570`
8. Zhang, C., Bengio, S., Hardt, M., Recht, B., Vinyals, O.: Understanding deep learning (still) requires rethinking generalization. Communications of the ACM **64**(3), 107–115 (2021)

---

[1] `https://www.editorialmanager.com/joms/default.aspx`

[2] LaTeX `https://static.springer.com/sgw/documents/468198/application/zip/LaTeX_DL_468198_01072021.zip`

[3] MS Word `https://static.springer.com/sgw/documents/431298/application/zip/sv-journ.zip`
